# Effects and feasibility of a preventive intervention in sub-threshold and mild panic disorder: Results of a pilot study

**DOI:** 10.1186/1756-0500-2-4

**Published:** 2009-01-09

**Authors:** Peter Meulenbeek, Godelief Willemse, Filip Smit, Niels Smits, Anton van Balkom, Philip Spinhoven, Pim Cuijpers

**Affiliations:** 1 Department of Clinical Psychology and EMGO Institute, VU-University, Amsterdam, The Netherlands; 2 GGNet, Community Mental Health Centre, Warnsveld, The Netherlands; 3 Trimbos instituut (Netherlands Institute of Mental Health and Addiction), Utrecht, The Netherlands; 4 Department of Psychiatry and EMGO Institute, VU-University Medical Centre, Amsterdam, The Netherlands; 5 Institute of Psychological Research, Unit of Clinical Psychology and Department of Psychiatry, Leiden University Medical Centre, Leiden University, Leiden, The Netherlands

## Abstract

**Background:**

Panic disorder (PD) is a serious DSM-IV axis I disorder affecting up to 3% of the adult population each year. It is associated with a large burden of disease and extensive economic costs. This study aims to examine the effects and feasibility of the 'Don't Panic' course, a preventive cognitive behavioural intervention in sub-threshold and mild PD. It also compares the effectiveness of two modifications of the course (8 vs. 12 sessions).

**Methods:**

The method used was a quasi-experimental two-group pre-post design with a baseline measurement (T0) and two follow-up measurements. Follow-ups were at the end of the intervention (T1) and six months later (T2). Primary outcome measure was the Panic Disorder Severity Scale-Self Report. A total of 114 participants suffering from panic attacks (mean age 42 years; 78% female) entered the study.

**Results:**

The course participants showed a significant effect on the outcome measures at follow-up. Large effect sizes were found on panic symptoms, on symptoms of agoraphobia and on mental health-related quality of life at T1 and T2. Overall, the course leaders and the participants evaluated the course positively. There were no significant differences in outcome measures between the short and the long version of the course.

**Conclusion:**

The study suggests that people with sub-threshold PD and mild PD could benefit from this preventive intervention and that the intervention might be feasible. Furthermore, the short version could be as effective as the long version.

## Background

Panic disorder (PD), affecting 2% to 3% of the adult population each year [1,2], is associated with a large burden of disease, considerable medical consumption and extensive loss of productivity [3,4]. The incidence of PD is high (about 35% of all cases each year of PD are new; [1]), indicating the importance of prevention and early intervention in PD.

A substantial proportion of the population suffers from sub-threshold PD [5,6]. Sub-threshold PD can be defined as the presence of some symptoms of PD, not meeting the DSM-IV diagnostic criteria. These subjects may be at risk of developing full-blown PD [7].

Studies on prevention and early intervention in anxiety disorders indicate that prevention of anxiety disorders through cognitive-behavioural interventions can be successful [8,9]. Only a few studies have been conducted on the prevention of panic disorder [10,11]. The results of these studies suggest that prevention of panic disorder is a promising option.

As a pilot project we developed a preventive intervention for adults with panic symptoms, called the 'Don't Panic' course. The course is based on cognitive-behavioural principles and makes use of interventions that have appeared effective in the treatment of PD before [12,13].

The aim of this study was to examine the effects and feasibility of the 'Don't Panic' course in a sample of self-referred people suffering from panic attacks in an uncontrolled pilot study. In addition, the effectiveness of two modifications of the course (8 vs. 12 sessions) was compared.

## Methods

### Research design

We used a quasi-experimental two-group pre-post design with a baseline measurement (T0), a post-test measurement at the end of the course (T1) and a follow-up measurement, 6 months after the course (T2). The study was designed to mimic the Dutch health care system as naturalistically as possible in terms of participant recruitment and the manner in which intake, offering the intervention, and monitoring outcomes are conducted. Because the study simulates the usual practice in the Netherlands, an ethical approval was not necessary.

### Participants

Participants were recruited from the general population through advertisements in regional newspapers and information brochures at general practices. For screening, the standard procedures employed by the Community Mental Health Centres were used. Firstly, interested persons were given more information about the course and underwent initial screening by telephone to ascertain the presence of panic symptoms. Secondly, potential participants had a face-to-face clinical interview with a clinician (i.e., experienced psychologist) from a Community Mental Health Centre. The clinician used a list with inclusion and exclusion criteria. Depending on the impression formed by the clinicians, potential participants were included or excluded. Those included in the study were people with sub-threshold panic disorder or mild panic disorder with or without additional agoraphobic problems (i.e., low degree of symptom severity and little interference in work or social functioning). Exclusion criteria were severe PD (i.e., high degree of symptom severity and substantial interference in work or social functioning), other current psychiatric diagnoses or suicidality warranting treatment or likely to interfere with participation in the group course. People meeting one of the exclusion criteria were advised to seek regular treatment. If a participant used medication for anxiety or depression (e.g., anxiolytics or antidepressants), it was agreed not to change the medication while attending the course. The participants were receiving no other form of psychotherapy.

**Figure 1:**
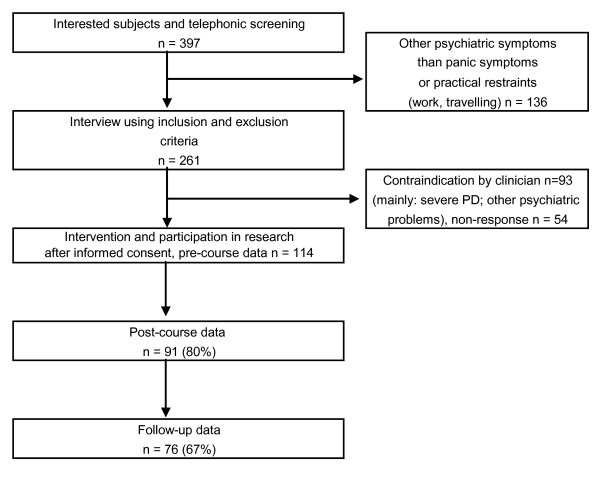
**Participants' flow through the study**. *Note*. PD = Panic Disorder.

For the participants' flow through the study see Figure 1. To join the study, the participant had to sign a written informed consent. Fifty-four participants refused to take part in the research (32%; mainly because they didn't feel like it), but were allowed to attend the course.

A total of 114 participants were enrolled in the study and completed the baseline measurements. In Table 1, the characteristics of the course participants are presented.

**Table 1:** Characteristics of the course participants for Total Group, Short Version and Long Version

	Total group (N = 114)	Long version (N = 78)	Short version (N = 36)
Female, N (%)	89 (78.1)	64 (82.1)	25 (69.4)
Mean age, Years (SD)	41.7 (11.4)	42.4 (11.4)	40.3 (11.5)
Married/living with partner, N (%)	87 (76.3)	58 (74.3)	29 (80.6)
Employed (paid), N (%)	85 (74.6)	56 (71.8)	29 (80.6)
Educational level, N (%)			
Low	22 (19.3)	15 (19.2)	7 (19.4)
Middle	51 (44.7)	36 (46.2)	15 (41.7)
High	41 (36.0)	27 (34.6)	14 (38.9)
More than 1 year of panic complaints, N (%)	95 (83.3)	65 (83.3)	30 (83.3)
Medication for anxiety or depression, N (%)	64 (56.1)	42 (53.8)	22 (61.1)
SCL-90 Anx, M (SD) (range: 0–4)	1.26 (0.72)	1.31 (0.76)	1.15 (0.63)
SCL-90 Pho, M (SD) (range: 0–4)	1.13 (0.83)	1.16 (0.84)	1.09 (0.81)
SCL-90 Dep, M (SD) (range: 0–4)	0.89 (0.62)	0.89 (0.63)	0.88 (0.61)
PDSS-SR, M (SD)* (range: 0–28)	8.7 (5.0)	8.8 (5.5)	8.6 (4.1)
Subclinical (score 0–7), N (%)	36 (40.4)	25 (47.2)	11 (30.6)
Clinical (score 8–28), N (%)	53 (59.6)	28 (52.8)	25 (69.4)

*Note*. SCL-90 = Symptom Check-List: Anx = anxiety, Pho = phobic anxiety, Dep = depression; PDSS-SR = Panic Disorder Severity Scale-Self Report.

* based on n = 89 (the PDSS-SR was not used on the first 3 courses).

A comparison of demographic and clinical status variables of the participants at baseline (T0) between the short (N = 36) and the long (N = 78) version of the course demonstrated no significant differences.

### Measures

Primary outcome measure for panic and agoraphobic symptoms was the self-report version of the Panic Disorder Severity Scale [14]. The Panic Disorder Severity Scale-Self Report (PDSS-SR) has good psychometric properties (Cronbach's alpha = 0.92; intraclass correlation coefficient = 0.81) [15]. Secondary outcomes for panic symptoms consisted of the Body Sensation Questionnaire (BSQ1; [16]) and the subscale *anxiety *of the Symptom Check-List (SCL-90; [17]). For symptoms of agoraphobia the secondary outcomes were the Agoraphobic Cognitions Questionnaire (ACQ; [16]) and the Mobility Inventory (MI; [18]). In addition, the subscale *phobic anxiety *of the SCL-90 [17] was used. For mental health-related quality of life, the outcome measure was the subscale *mental health *of the MOS Short-Form General Health Survey (MOS-SF-20 Mh; [19]). To measure general symptoms of depression, the subscale *depression *of the SCL-90 [17] was used. Moreover, the Mastery-scale [20] was used to assess locus of control. All scales have good psychometric properties [16-20]. Only on the MOS-SF-20 MH and the Mastery-scale does a higher rating indicate a more favourable outcome.

Questionnaires to evaluate the course were used at post-test.

### Intervention

The 'Don't Panic' course includes a course manual [21], to be used by the clinician and prevention specialist offering the intervention, as well as a participant workbook [22]. The course involves (a) psycho-education about the nature and physiology of anxiety and panic attacks, (b) changing life-style to enhance physical condition, (c) stress management to prevent constant stress by learning effective ways to cope with stressors, (d) relaxation training to reduce physiological arousal, (e) cognitive restructuring to challenge and correct faulty beliefs regarding panic and anxiety, (f) interoceptive exposure to reduce the fear of somatic sensations, and (g) 'in vivo' exposure to reduce avoidance of situations and use of safety behaviours. Furthermore, techniques aimed at relapse prevention are taught. The short course consists of 8 weekly, two-hour group sessions; the long version contains 12 weekly, two-hour group sessions of 6 to 12 participants. Both versions have the same elements. The long version has more sessions containing 'in vivo' exposure.

### Procedure

Clinicians and prevention specialists from 12 community mental health centres were trained in the selection procedure of participants (e.g., using the inclusion and exclusion criteria) and in using the course manual to conduct the course. Integrity of intervention delivery was maintained by utilizing a structured and manual-based course protocol.

The twelve-session course was run by 9 centres and the 8 session course by 6 centres (3 centres offered both versions of the course; one mental health centre offered the long version of the course four times. In these cases the time between two courses was about two months and a person participated in the course offered at the time the person entered the study. There was no selection of participants to the two versions of the course.). In total, the data contains the results of 18 courses.

Data collection was conducted by an independent research institute. Assessments consisted solely of self-report measures through postal questionnaires and were completed at home.

### Statistical analyses

Analyses were conducted on an intention-to-treat basis. The effect of the intervention was analysed with a Repeated Measures ANOVA, Simple Contrast. Effect sizes (*d*) were obtained by subtracting post-test (T1) or follow-up (T2) means from baseline (T0) means and then dividing the difference by the pooled standard deviation. The categorization of Lipsey and Wilson [23] was used for clinical interpretations of the *d*'s.

The difference between the short and the long version of the course on the severity of panic symptoms was analysed with a 2 Group (8 vs. 12 sessions) × 3 Time (baseline, post-test, 6 month follow-up) Repeated Measures ANOVA, with Group as the between-subject factor and Time as within-subject factor. Levels of severity of panic symptoms (PDSS-SR) constituted the dependent variable. Wilks' lambda (λ) was used as multivariate criterion for significance. The same analysis was performed on all the other outcome measurements.

The significance level was set at α = 0.05, two-sided.

## Results

### Outcome

Table 2 presents an overview of the results on the measurements. The course participants showed a significant effect on the primary outcome measure and secondary outcome measures at post-test and follow-up. Large effect sizes were found on panic symptoms and on symptoms of agoraphobia at T1 and T2. In addition, large effect sizes were found on mental health-related quality of life at T1 and T2 and on depression at T2. The increase of internal locus of control after the intervention showed medium effect sizes. Effects remained stable from post-test to follow-up.

**Table 2:** Means, Standard Deviations, Repeated Measures ANOVA, Simple Contrast and (range) Effect Sizes (*d*) for Baseline (T0), Post-test (T1), and Follow-up (T2) (N = 114)

Scale measure	T0 Mean	(SD)	T1 Mean	(SD)	T2 Mean	(SD)	Effect Size (d) T0-T1	Range (d) (T0-T1)	Effect Size (d) T0-T2	Range (d) (T0-T2)
PDSS-SR*	8.74	(4.98)	5.03**	(4.98)	5.07**	(5.39)	0.75	-1.9–2.8	0.71	-3.1–3.2
(range: 0–28)										
BSQ1	2.04	(0.62)	1.47**	(0.49)	1.42**	(0.60)	1.02	-1.0–5.3	1.02	-1.7–3.6
(range: 1–5)										
ACQ	2.00	(0.66)	1.47**	(0.60)	1.42**	(0.56)	0.84	-0.8–3.6	0.95	-1.5–3.1
(range: 1–5)										
MI	2.15	(0.78)	1.65**	(0.73)	1.61**	(0.60)	0.66	-1.6–3.5	0.77	-2.8–3.5
(range: 1–5)										
SCL-90 Anx	1.26	(0.72)	0.68**	(0.64)	0.62**	(0.60)	0.84	-1.1–4.4	0.96	-2.1–4.4
(range: 0–4)										
SCL-90 Pho	1.13	(0.83)	0.63**	(0.72)	0.50**	(0.60)	0.64	-1.7–3.9	0.88	-2.1–3.9
(range: 0–4)										
SCL-90 Dep	0.89	(0.62)	0.56**	(0.59)	0.51**	(0.57)	0.55	-1.2–3.2	0.63	-3.8–3.4
(range: 0–4)										
MOS-SF-20 Mh	57.60	(16.43)	67.95**	(17.09)	68.76**	(18.38)	0.62	-1.7–2.7	0.64	-1.5–2.9
(range: 0–100)										
MASTERY*	17.47	(4.21)	18.91**	(3.93)	19.11**	(4.32)	0.35	-1.6–3.1	0.38	-1.8–2.7
(range: 5–25)										

*Note*. PDSS-SR = Panic Disorder Severity Scale-Self Report; BSQ1 = Body Sensation Questionnaire; ACQ = Agoraphobic Cognitions Questionnaire, total score; MI = Mobility Inventory, total score; SCL-90 = Symptom Check-List: Anx = anxiety; Pho = phobic anxiety; Dep = depression; MOS-SF-20 = Medical Outcomes Study Short-Form General Health Survey (SF-20): Mh = mental health.

* based on n = 89 (the PDSS-SR and MASTERY were not used on the first 3 courses).

** Repeated Measures ANOVA, Simple Contrast p < 0.001.

The Repeated Measures ANOVAs on the outcome measures showed a significant effect of time, no significant group effect between the short and the long version of the course and no significant interaction effect for time × group (Table 3).

**Table 3:** Repeated Measures ANOVA on the outcome measures by group (N = 114)

Scale	Intervention (time) effect	Group (condition) effect	Interaction effect for intervention × group
measure	*F *and *p*	*F *and *p*	*F* and *p*
PDSS-SR*	*F *(2, 86) = 28.321, *p *= <0.001	*F *(1, 87) = 0.086, *p *= 0.861	*F *(2, 86) = 0.112, *p *= 0.926
BSQ1	*F *(2, 111) = 66.789, *p *= <0.001	*F *(1, 112) = 0.059, *p *= 0.820	F (2, 111) = 0.899, *p *= 0.612
ACQ	*F *(2, 111) = 58.273, *p *= <0.001	*F *(1, 112) = 0.019, *p *= 0.914	F (2, 111) = 0.578, *p *= 0.734
MI	*F *(2, 111) = 39.805, *p *= <0.001	*F *(1, 112) = 0.415, *p *= 0.527	F (2, 111) = 0.926, *p *= 0.605
SCL-90 Anx	*F *(2, 111) = 45.702, *p *= <0.001	*F *(1, 112) = 0.148, *p *= 0.743	F (2, 111) = 1.206, *p *= 0.491
SCL-90 Pho	*F *(2, 111) = 33.398, *p *= <0.001	*F *(1, 112) = 0.084, *p *= 0.842	F (2, 111) = 0.722, *p *= 0.650
SCL-90 Dep	*F *(2, 111) = 22.132, *p *= <0.001	*F *(1, 112) = 0.082, *p *= 0.849	F (2, 111) = 0.411, *p *= 0.768
MOS-SF-20 Mh	*F *(2, 111) = 31.287, *p *= <0.001	*F *(1, 112) = 0.246, *p *= 0.646	F (2, 111) = 0.721, *p *= 0.633
MASTERY*	*F *(2, 86) = 11.400, *p *= <0.009	*F *(1, 87) = 2.054, *p *= 0.168	F (2, 86) = 0.453, *p *= 0.747

*Note*. PDSS-SR = Panic Disorder Severity Scale-Self Report; BSQ1 = Body Sensation Questionnaire; ACQ = Agoraphobic Cognitions Questionnaire, total score; MI = Mobility Inventory, total score; SCL-90 = Symptom Check-List: Anx = anxiety; Pho = phobic anxiety; Dep = depression; MOS-SF-20 = Medical Outcomes Study Short-Form General Health Survey (SF-20): Mh = mental health.

* based on n = 89 (the PDSS-SR and MASTERY were not used on the first 3 courses).

Medication use at baseline had no significant impact on the residualized change scores at T1 on the primary outcome measure (PDSS-SR; point-biserial correlation; *r *= 0.12, *p *= 0.249). For the other outcome measures at T1 (except for MOS-SF-20 Mh; point-biserial correlation; *r *= -0.20, *p *= 0.029) and for all outcome measures at T2, the results of the analyses were comparable.

### Feasibility and acceptability

After each session the prevention specialists registered all the participants attending, and ascertained whether they had done their homework. On the basis of this information, the dropout rate (participants who indicated stopping the course, mainly because of work obligations or illness) was 13%; the mean number of attended sessions for the short version was 6.4 sessions (80%) and for the long version 9.0 sessions (75%). Of the participants attending a session, on average 91% had completed their homework. Therefore, compliance with the intervention was satisfactory.

The participants evaluated the course positively (organizational aspects, coaching, content, group sessions, and workbook). Asked whether the course had contributed to being more able to manage anxiety, 98% answered in the affirmative. Of the participants, 92% were "satisfied" or "very satisfied" with the course.

Overall, the course leaders also evaluated the course positively (content, time schedule, didactic elements). These findings suggest the intervention is feasible and acceptable.

## Discussion

In this study, the participants in the preventive course 'Don't Panic' showed a significant reduction in panic and agoraphobic symptoms after the course. Large effect sizes were achieved in reducing panic and agoraphobic symptoms. The improvement was maintained over 6 months after the intervention. The results suggest that the course is acceptable and feasible. These findings converge with the findings of earlier research on the prevention of panic disorder [10,11].

Furthermore, symptoms of depression declined, as measured by the SCL-90. In addition, the internal locus of control and the quality of life increased significantly. These findings are important because people with panic symptoms may also have higher rates of other psychiatric symptoms [24].

We did not find any significant differences in outcome measures between the short and the long version of the course. A lack of power may play a role in this finding.

There are several limitations to the present study. Firstly, it lacked a structured psychiatric interview. Therefore, it is unclear how many participants might have been diagnosed with panic disorder or other psychiatric disorders at baseline, post-test and follow-up. Furthermore, the diagnosis of agoraphobia might have clarified whether it had an impact on the results.

Secondly, 56% of the participants used medication for anxiety or depression at baseline. Although medication use at baseline did not predict outcome, continued use of medication may have hampered the effect of the intervention. This is because medication may have been used as a safety behaviour. Likewise, discontinuation of medication after psychological treatment may also have reduced treatment efficacy because patients may attribute the symptom reduction achieved to the use of medication and may lack self-confidence in being able to control their panic attacks themselves. In the absence of data on medication use after baseline assessment, the possible interactions of medication use with psychological treatment remain unknown.

Thirdly, the present study did not include a control group. Thus, it is not certain whether the effects found in this study were due to the preventive course.

Fourthly, the study did not account for possible differences in outcome between the 12 community mental health centres which offered the course.

Fifthly, because the intervention is multi-component, it cannot be known which of the components may have been effective.

Finally, the results may be biased if people of the intended target population who did not participate in the course or participated in the course, but not in the study, differed from those who did.

## Conclusion

People with sub-threshold PD and mild PD may benefit from a preventive group intervention. The short version is as effective as the long version, but is likely to be associated with fewer costs and greater acceptability. Future, randomized, controlled trials using diagnostic assessments are needed to confirm the present findings.

## Competing interests

The authors declare that they have no competing interests.

## Authors' contributions

PM participated in the design of the study, conducted the analysis and drafted the manuscript. GW participated in the design of the study, collected the data and participated in the analysis of the data. FS participated in the design of the study and in the analysis of the data. NS participated in the statistical analysis of the data. PC, AvB and PS acted as a Quality Assurance Committee for this trial. All authors were involved in the interpretation of the data and approved the final manuscript.
